# Microcrystal manipulation with laser tweezers

**DOI:** 10.1107/S090744491300958X

**Published:** 2013-06-13

**Authors:** Armin Wagner, Ramona Duman, Bob Stevens, Andy Ward

**Affiliations:** a Diamond Light Source, Harwell Science and Innovation Campus, Chilton, Didcot OX11 0DE, England; b School of Science and Technology, Nottingham Trent University, Nottingham NG1 4BU, England; c Central Laser Facility, STFC, Research Complex at Harwell, Didcot OX11 0FA, England

**Keywords:** laser tweezers, optical trapping, microcrystals, crystal manipulation, sample holders

## Abstract

X-ray crystallography is the method of choice to deduce atomic resolution structural information from macromolecules. In recent years, significant investments in structural genomics initiatives have been undertaken to automate all steps in X-ray crystallography from protein expression to structure solution. Robotic systems are widely used to prepare crystallization screens and change samples on synchrotron beamlines for macromolecular crystallography. The only remaining manual handling step is the transfer of the crystal from the mother liquor onto the crystal holder. Manual mounting is relatively straightforward for crystals with dimensions of >25 µm; however, this step is nontrivial for smaller crystals. The mounting of microcrystals is becoming increasingly important as advances in microfocus synchrotron beamlines now allow data collection from crystals with dimensions of only a few micrometres. To make optimal usage of these beamlines, new approaches have to be taken to facilitate and automate this last manual handling step. Optical tweezers, which are routinely used for the manipulation of micrometre-sized objects, have successfully been applied to sort and mount macromolecular crystals on newly designed crystal holders. Diffraction data from CPV type 1 polyhedrin microcrystals mounted with laser tweezers are presented.

## Abstract 2

Optical trapping has successfully been applied to select and mount microcrystals for subsequent X-ray diffraction experiments.

## 1. Introduction

Recent developments in synchrotron hardware (Perrakis *et al.*, 1999 ▶; Flot *et al.*, 2006 ▶; Evans *et al.*, 2007 ▶; Kunio *et al.*, 2010 ▶; Fischetti *et al.*, 2009 ▶) allow macromolecular crystallography experiments to be routinely performed using crystals with dimensions smaller than 10 µm. A major problem encountered when working with microcrystals is that their transfer to sample holders becomes increasingly more difficult as the crystal size decreases. Microcrystals can be grown in populations of hundreds, from which it is almost impossible to manually separate individual crystals, select the largest ones and then mount them on existing sample mounts. Therefore, in many cases a random approach is adopted in which a sample mount is swept through a droplet containing a dispersion of microcrystals. The success of this method can only be evaluated on the beamline and no control over the process is usually possible. A technique that permits the pre-selection of samples would allow optimal usage of the available beam time at dedicated microfocus beamlines.

To overcome this limitation, several approaches can be taken. Soares and coworkers have recently demonstrated the successful application of an acoustic droplet injector to transfer microcrystals from a crystallization drop onto standard sample holders (Soares *et al.*, 2011 ▶). Piezo-electric grippers with submicrometre positioning accuracy are used by G-­Rob (Jacquamet *et al.*, 2004 ▶). After harvesting crystals directly from crystallization plates and freezing, the robot is used as a goniometer to perform the X-ray diffraction experiment. CrystalDirect (Cipriani *et al.*, 2012 ▶), a project at EMBL Grenoble, uses a different approach. Crystals are grown on a thin film and a focused laser beam is used to cut out the region of interest containing the crystal. This avoids any crystal manipulation, as the thin film acts as a sample holder during data collection.

Here, we present the successful application of optical traps or laser tweezers for microcrystal manipulation and mounting. Laser tweezers have become a routine method to manipulate micrometre-sized objects and the technique is widely used throughout a variety of life-science applications such as cell sorting, as well as for the measurement of small forces in the piconewton range. Optical trapping was first described by Ashkin (1970 ▶) and a variety of reviews that describe the underlying physics and applications have been published (Svoboda & Block, 1994 ▶; Ashkin, 1997 ▶). In brief, a single-beam gradient trap is formed by a highly focused laser beam from an objective lens with a high numerical aperture (NA). The challenge for laser tweezers-based manipulation of microcrystals is twofold. The first is to determine whether crystalline geometries can be captured with a sufficient trapping force to permit mounting of the crystal onto a sample holder, while the second challenge is to use laser powers that do not cause photothermal and photochemical degradation of the crystal structure. The single-beam gradient trap is based on the equilibrium between the so-called gradient forces arising from refraction and radiation pressure from laser scattering when a particle of refractive index greater than the surrounding media is at the focal point of the laser beam. A laser-tweezers system for microcrystal handling under develop­ment at SPring-8 (Kunio *et al.*, 2010 ▶) uses a slightly different approach. It is based on two optical fibres at an angle providing an optical trap independent of the visualization system (Taguchi *et al.*, 2000 ▶).

## 2. Experimental

### 2.1. Experimental setup

For initial studies, the experimental setup at the Central Laser Facility was used. This system comprised a Leica DM-IRB inverted microscope with a water-immersion objective lens of NA 1.2. Two laser systems were used for optical trapping: an Ar-ion laser at 514.5 nm (Coherent) and a near-infrared Nd:YAG laser at 1064 nm (Laser Quantum). The laser power used for optical trapping could be varied, but was typically less than 50 mW at the focal spot.

A motorized sample stage (0.05 µm resolution) allowed movement of the liquid sample droplet containing crystals relative to the fixed optical system. The stage was used to screen the drop for crystals prior to trapping and to position candidate crystals into the trapping field. A trapped crystal could be moved relative to the sample by translating the stage at velocities of up to 200 µm s^−1^. A greater velocity caused viscous drag forces to dislodge the crystals. Changing the distance between the objective lens and the cover slip enabled vertical movement of the trapped crystals. Additionally, several optical traps could be produced and moved independently within the microscope field of view by changing the incident angle of the laser into the microscope objective lens using acousto-optic deflection. Manual micromanipulators were used to allow independent movement of the sample holders relative to the optical trapping position and the microscope stage.

**Figure 1:**
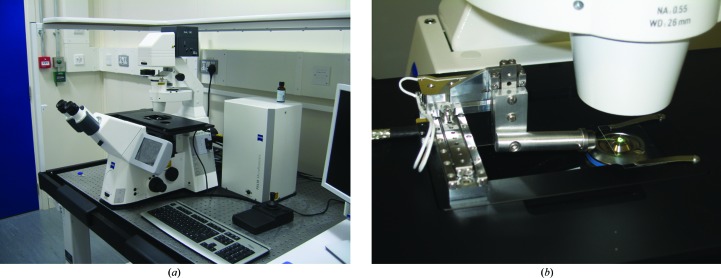
PALM MicroTweezers microscope (Zeiss). (*a*) Overall view. (*b*) Sample environment with SmarAct micromanipulators for sample holders.

Based on successful pre-studies at the Central Laser Facility, a PALM MicroTweezers system was purchased. It is based on the Zeiss AxioObserver Z1 inverted microscope and, in collaboration with ZeissMicroimaging, a set of micromanipulators was integrated on the microscope sample stage (Fig. 1 ▶). Coupling the micromanipulators with the sample stage allowed a fixed loading position within the drop and avoided additional flow effects when screening a large field of view. The Zeiss optical trapping system comprises a 63× water-immersion objective lens (NA 1.2) and a 1.5 W 1064 nm Nd:YAG laser. Owing to the microscope design, no additional safety measures are needed during sample manipulation and mounting. The laser can be split into two independent traps which can act on different parts of a crystal. The control of the laser trap is integrated into the microscope software, which also allows access to all relevant parameters for stage movement. A very useful feature, facilitating the movement of crystals in the droplet, is the option to save positions and define trajectories in between markers which can be tracked either by the laser trap or the microscope stage.

### 2.2. Crystals

Throughout the tests the following well characterized microcrystals were used. Cubic cypovirus (CPV) polyhedrin type 1 crystals (Coulibaly *et al.*, 2007 ▶) were obtained as cubes of dimensions of between 2 × 2 × 2 and 12 × 12 × 12 µm. They were used in the sorting, mounting and diffraction experiments. They grow in the cubic space group *I*23, with unit-cell parameter *a* = 102.75 Å. Additionally, for crystal-mounting experiments hexagonal bipyramidal Ultralente insulin crystals (25 × 25 × 5 µm; Wagner *et al.*, 2009 ▶) were chosen to test the applicability of the laser-trapping method for larger plate-like crystals. Both crystals could be obtained in large quantities and therefore represent ideal test systems for microcrystal work.

### 2.3. Sample holders

Different types of commercially available sample holders were tested for their laser compatibility. No visible damage to nylon CryoLoops could be observed at the two wavelengths; however, because of the large aperture (>50 µm) these sample holders were not considered further. LithoLoops (Molecular Dimensions) showed strong fluorescence and visible radiation damage in the Ar-ion laser and instantaneous heating manifested as bubbling in both laser beams even at low laser power (<50 mW). The laser damage appeared to arise from inclusions and imperfections in the loops which were visible on the microscopic scale. Better results were obtained using micromeshes (MiTeGen). These Kapton meshes showed radiation damage in the Ar-ion laser beam, but could withstand over 100 mW laser power at 1064 nm. They are essentially transparent to the IR laser, and any optical aberration of the focused trapping beam was insufficient to perturb the optical trapping stability except in a region of 10 µm above the grid. Therefore, for all subsequent crystal-mounting experiments, and for the experimental modifications described below, only these micromeshes in combination with IR lasers were considered further.

The main problem with the micromeshes was removing them from the liquid droplet reliably without the crystals dropping through the voids in the mesh. For this reason, we investigated different methods to provide meshes with a ‘floor’ to prevent crystals from falling through the holes and to allow sufficient adhesion for the crystals to stay in position whilst the micromesh was extracted from the sample drop.

**Figure 2:**
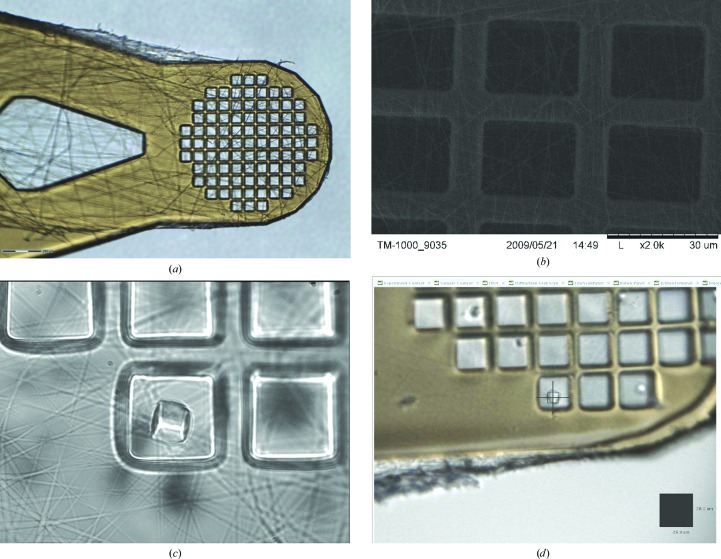
Micromeshes (25 µm openings) with PMMA fibres. (*a*) 10× optical microscope. (*b*) TEM. (*c*) A CPV polyhedrin crystal on the laser-tweezers microscope. (*d*) The same CPV polyhedrin crystal on the I24 on-axis viewing system (behind the cross-hair representing the beam position).

The best results were achieved by electrospinning a thin nanofibre non-woven mesh of 150 nm diameter poly(methyl methacrylate) (PMMA) over the Kapton micromesh. The electrospinning parameters, which include the temperature and humidity of the electrospinning environment (Hardick *et al.*, 2011 ▶), were investigated to produce PMMA nanofibres with a reproducible diameter. Micromeshes (MiTeGen) were mounted on a stainless-steel pin and placed on the cathode electrode of the electrospinning system. The nanofibre network was deposited and covered the holder and the Kapton mesh of the micromesh pins. A hot blade was used to cut the nanofibre mat in order to allow each coated micromesh pin to be removed. An optical image and a transmission electron-microscopy image of the PMMA fibres on a micromesh are shown in Figs. 2 ▶(*a*) and 2 ▶(*b*), respectively.

## 3. Results and discussion

### 3.1. Optical trapping

**Figure 3:**
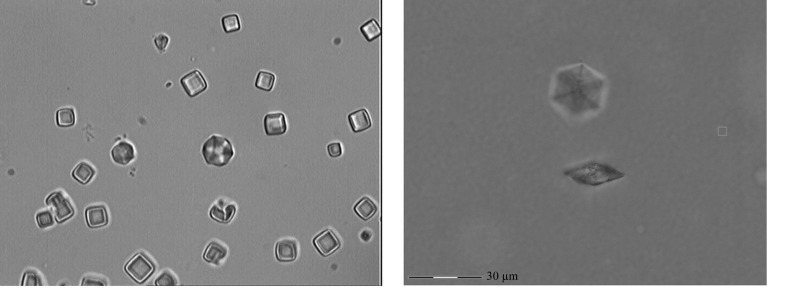
Trapped microcrystals. (*a*) CPV polyhedrin. (*b*) Ultralente.

Optical trapping was straightforward for the smaller CPV polyhedrin cubes and was feasible for most of the Ultralente crystals. Trapped crystals instantaneously oriented themselves with the longest axis along the laser-beam axis, which resulted in the corners of the cubes and the Ultralente plates pointing upwards (Fig. 3 ▶). A 10 µm CPV polyhedrin crystal could be held with a laser power of 50 mW when moving the stage and surrounding environment at 200 µm s^−1^; this approximates to an 18 pN trapping force. For some crystals the trapping force was not sufficient to overcome surface adhesion between the crystals and the cover slip. In particular, an increase in the concentration of cryoprotectants, such as ethylene glycol or glycerol, was found to negatively affect the likelihood of adhesion. An influence of the cover-slip coating could also be observed, but more systematic studies are necessary to understand the effects of these coatings. However, many crystals could be released by gently tapping the cover slip to overcome adhesion.

### 3.2. Crystal sorting and mounting

Crystals could easily be selected by size and sorted in predefined regions within the sample drop analogously to Huang *et al.* (2009 ▶). After trapping, the crystals were lifted between 30 and 50 µm above the surface. To move the crystal within the drop, the sample stage was moved relative to the fixed optical trap.

For crystal loading onto a sample holder, a droplet containing a dilution of crystals and cryoprotectant was placed on a cover slip and crystals were allowed to settle by gravity to the bottom of the cover slip. A sample holder was selected and wetted with a droplet of mother liquor to prevent residual air bubbles on the mesh. It was subsequently mounted onto the micromanipulator and carefully manoeuvred through the droplet–air interface. The sample holder was positioned using the optical imaging of the microscope to a depth of approximately 30 µm above the cover slip. A marker was set in the Zeiss microscope software to mark the loading position close to the mesh. To find a suitable crystal, a scan over a large area was defined and the images were stitched together by the software. Markers were set at the positions of large crystals. A selected crystal was then trapped, lifted to initially 25 µm above the cover slip and automatically moved to the loading position. For final loading and placement on the mesh, the crystal was lifted to around 50 µm above the cover slip, slowly moved across the mesh and then lowered into the mesh openings.

The main difficulty in crystal mounting is that the crystal has to be trapped and held during this step. The edge of the mesh, and the change in refractive index, can affect the optical trap properties. For the smaller CPV polyhedrin crystals no severe effects were observed. However, for the larger Ultralente crystals with their greater mass and plate-like shape the edge crossing was more difficult. Only when lifted around 50 µm above the mesh surface were the changes in the optical trap small enough to avoid losing the crystal.

After mounting four to six crystals on a mesh, the micromanipulators were used to slowly remove the sample holder from the droplet, keeping the solvent film on the mesh to less than 10 µm. The sample holder was then quickly removed from the magnetic interface to the micromanipulator and flash-cooled in liquid nitrogen.

### 3.3. X-ray diffraction experiments

X-ray diffraction experiments were performed with laser-mounted CPV polyhedrin crystals on the microfocus macromolecular crystallography beamline I24 at Diamond Light Source using a focused beam of 7 × 7 µm (FWHM) at a wavelength of λ = 0.9795 Å. Only cubes with edges larger than 10 µm were mounted. Complete diffraction data to a resolution of 1.5 Å could be obtained from a single crystal of 11 × 11 × 11 µm in size (Figs. 2 ▶ *c* and 2 ▶ *d*). Data-collection parameters can be found in Table 1 ▶.

**Figure 4:**
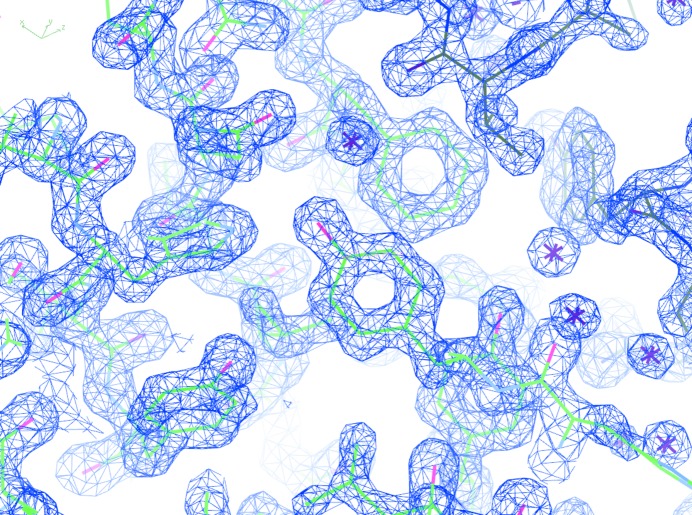
Representative part of the 1.5 Å resolution electron-density map for CPV polyhedrin (2*F*_o_ − *F*_c_, 1.3σ).

Diffraction data were processed with *XDS* (Kabsch, 2010 ▶) and the structure was solved by molecular replacement with the published CPV polyhedrin protein structure model (PDB entry 2oh6; Coulibaly *et al.*, 2007 ▶) using *Phaser* (McCoy *et al.*, 2007 ▶). The structure was refined with *PHENIX* (Adams *et al.*, 2010 ▶) to *R*_work_ = 14.8% and *R*_free_ = 17.8%. Fig. 4 ▶ shows a representative part of the electron density.

### 3.4. Current limitations

The main limitation of the current implementation of the technique is that the objective is corrected for 0.14–0.19 mm thick cover slips. Crystals need to be either transferred on such a cover slip or directly grown on them by the hanging-drop vapour-diffusion method. The inverted microscope geometry allows access from the top and the high numerical aperture gives very high magnification. At present, the sample environment is not humidity-controlled, which requires the use of large drops containing crystals to allow sufficient time for manipulation.

The current crystal-mounting procedure is based on a combination of automated and manual steps. Scanning the drop containing crystals and stitching the resulting images to identify the locations of the larger crystals is part of the microscope software. Tests with additional image-processing modules showed promising results in automatic recognition of the largest crystals and are considered as a potential upgrade. In cases when the crystals stick to the cover-slip surface the initial trapping still needs to be manual, while moving trapped crystals to a pre-defined position along a trajectory can be automated. During the final approach onto the mesh and final placement of the crystal the software has to be operated through user inputs to guide the crystal to its final loading position.

The laser power is a critical parameter when trapping crystals. At full laser power the crystals can be easily trapped. However, they lose their diffraction properties without visual damage. We typically operate with laser powers below 120 mW, which is a good compromise between ease of manipulation and prevention of laser damage.

## 4. Conclusion

Several synchrotron beamlines routinely allow focused beam sizes smaller than 10 µm, and projects to provide submicrometre beam sizes are currently in the planning and design phase. For efficient usage of these beamlines, the handling of microcrystals is becoming a major challenge. Laser tweezers can overcome this limitation as they allow the manipulation of objects smaller than 25 µm; in fact, the smaller the objects the easier the manipulation. The 63× water-immersion objective of the laser-tweezers microscope has a resolution of around 250 nm for visible light, which is significantly better than the resolutions obtained from stereomicroscopes which are typically used for crystal inspection and mounting. This superior visualization allows the identification of crystal defects and the selection of individual crystals, *e.g.* the largest within a given sample.

The ability to choose the largest crystals from a suspension of thousands of smaller CPV polyhedrin crystals using laser tweezers enabled us to obtain significantly better quality diffraction data compared with the original publication (Coulibaly *et al.*, 2007 ▶). Data to a resolution of 1.5 Å could be obtained with a single CPV polyhedrin crystal from a total of five screened in a single session, whilst more than 100 crystals had to be tested for the 2.1 Å resolution original structure, which was based on merged data from two crystals.

The development of the sample holders turned out to be the most critical step in the project. Several alternative routes based on diamond and silica structures failed. Electrospinning of nanofibres in combination with established commercially available mounts worked and will be further investigated as it has great potential to facilitate manual crystal mounting in general.

The presented crystal-mounting technique is aimed towards projects for which not the amount of crystals but their size is the limiting factor. Typically, the number of crystals is inversely proportional to the crystal size. Currently, large investments are being made in nanocrystallography experiments at free-electron laser facilities, where at present thousands to millions of crystals of a size of up to 2 µm are still needed per data set (Chapman *et al.*, 2011 ▶). Microcrystal mounting with laser tweezers can help to fill the size gap between standard macromolecular crystallography at synchrotrons and these new developments, by providing a tool for a more selective method for crystals in the size range from 2 to 10 µm, to facilitate structural studies at third-generation synchrotron sources.

## Figures and Tables

**Table 1:** Data-collection statistics Values in parentheses are for the highest resolution shell.

Wavelength (Å)	0.97
Space group	*I*23
Unit-cell parameters (Å, °)	*a* = *b* = *c* = 102.75, α = β = γ = 90
Resolution (Å)	60–1.5 (1.6–1.5)
*R*_merge_ (%)	11.4 (62.2)
CC_1/2_	99.5 (68.6)
〈*I*/σ(*I*)〉	8.07 (2.40)
Completeness (%)	97.8 (98.2)
No. of reflections	104635 (16236)
No. of unique reflections	28352 (4943)
Refinement: *R*_free_/*R*_work_ (%)	14.8/17.8

## References

[bb1] Adams, P. D. *et al.* (2010). *Acta Cryst.* D**66**, 213–221.

[bb2] Ashkin, A. (1970). *Phys. Rev. Lett.* **24**, 156–159.

[bb3] Ashkin, A. (1997). *Proc. Natl Acad. Sci. USA*, **94**, 4853–4860. 10.1073/pnas.94.10.4853 PMC24595 9144154

[bb4] Chapman, H. N. *et al.* (2011). *Nature (London)*, **470**, 73–77.

[bb5] Cipriani, F., Röwer, M., Landret, C., Zander, U., Felisaz, F. & Márquez, J. A. (2012). *Acta Cryst.* D**68**, 1393–1399. 10.1107/S0907444912031459 22993093

[bb6] Coulibaly, F., Chiu, E., Ikeda, K., Gutmann, S., Haebel, P. W., Schulze-Briese, C., Mori, H. & Metcalf, P. (2007). *Nature (London)*, **446**, 97–101. 10.1038/nature05628 17330045

[bb7] Evans, G., Alianelli, L., Burt, M., Wagner, A. & Sawhney, K. J. S. (2007). *AIP Conf. Proc.* **879**, 836–839.

[bb8] Fischetti, R. F., Xu, S., Yoder, D. W., Becker, M., Nagarajan, V., Sanishvili, R., Hilgart, M. C., Stepanov, S., Makarov, O. & Smith, J. L. (2009). *J. Synchrotron Rad.* **16**, 217–225. 10.1107/S0909049508040612 PMC2725011 19240333

[bb9] Flot, D., Gordon, E. J., Hall, D. R., Leonard, G. A., McCarthy, A., McCarthy, J., McSweeney, S., Mitchell, E., Nurizzo, D., Ravelli, R. G. B. & Shepard, W. (2006). *Acta Cryst.* D**62**, 65–71. 10.1107/S0907444905032646 16369095

[bb10] Hardick, O., Stevens, B. & Bracewell, D. (2011). *J. Mater. Sci.* **46**, 3890–3898.

[bb11] Huang, W. E., Ward, A. D. & Whiteley, A. S. (2009). *Env. Microbiol. Rep.* **1**, 44–49. 10.1111/j.1758-2229.2008.00002.x 23765719

[bb12] Jacquamet, L., Ohana, J., Joly, J., Legrand, P., Kahn, R., Borel, F., Pirocchi, M., Charrault, P., Carpentier, P. & Ferrer, J.-L. (2004). *Acta Cryst.* D**60**, 888–894. 10.1107/S0907444904005232 15103134

[bb13] Kabsch, W. (2010). *Acta Cryst.* D**66**, 125–132. 10.1107/S0907444909047337 PMC2815665 20124692

[bb14] Kunio, H., Go, U., Atsushi, N., Yoshiaki, K., Takaaki, H., Nobutaka, S., Takashi, K., Hirokatsu, Y., Takashi, T., Sunao, T., Kunikazu, T., Haruhiko, O., Shunji, G., Hideo, K. & Masaki, Y. (2010). *AIP Conf. Proc.* **1234**, 901–904.

[bb15] McCoy, A. J., Grosse-Kunstleve, R. W., Adams, P. D., Winn, M. D., Storoni, L. C. & Read, R. J. (2007). *J. Appl. Cryst.* **40**, 658–674. 10.1107/S0021889807021206 PMC2483472 19461840

[bb16] Perrakis, A., Cipriani, F., Castagna, J.-C., Claustre, L., Burghammer, M., Riekel, C. & Cusack, S. (1999). *Acta Cryst.* D**55**, 1765–1770. 10.1107/s0907444999009348 10531527

[bb17] Soares, A. S., Engel, M. A., Stearns, R., Datwani, S., Olechno, J., Ellson, R., Skinner, J. M., Allaire, M. & Orville, A. M. (2011). *Biochemistry*, **50**, 4399–4401. 10.1021/bi200549x PMC3144476 21542590

[bb18] Svoboda, K. & Block, S. M. (1994). *Annu. Rev. Biophys. Biomol. Struct.* **23**, 247–285. 10.1146/annurev.bb.23.060194.001335 7919782

[bb19] Taguchi, K., Atsuta, K., Nakata, T. & Ikeda, M. (2000). *Opt. Commun.* **176**, 43–47.

[bb20] Wagner, A., Diez, J., Schulze-Briese, C. & Schluckebier, G. (2009). *Proteins*, **74**, 1018–1027. 10.1002/prot.22213 18767151

